# The Method of Silent Substitution for Examining Melanopsin Contributions to Pupil Control

**DOI:** 10.3389/fneur.2018.00941

**Published:** 2018-11-27

**Authors:** Manuel Spitschan, Tom Woelders

**Affiliations:** ^1^Department of Experimental Psychology, University of Oxford, Oxford, United Kingdom; ^2^Chronobiology Unit, Groningen Institute for Evolutionary Life Sciences, University of Groningen, Groningen, Netherlands

**Keywords:** pupil, melanopsin, silent substitution, color vision, pupillometry, ipRGC (intrinsically photosensitive retinal ganglion cell), metamers

## Abstract

The human pupillary light response is driven by all classes of photoreceptors in the human eye—the three classes of cones, the rods, and the intrinsically photosensitive retinal ganglion cells (ipRGCs) expressing the photopigment melanopsin. These photoreceptor classes have distinct but overlapping spectral tuning, and even a monochromatic light with a wavelength matched to the peak spectral sensitivity of a given photoreceptor will stimulate all photoreceptors. The method of silent substitution uses pairs of lights (“metamers”) to selectively stimulate a given class of photoreceptors while keeping the activation of all others constant. In this primer, we describe the method of silent substitution and provide an overview of studies that have used it to examine inputs to the human pupillary light response.

## Introduction

At the input level, the size of the pupil is controlled by the activity of the different photoreceptors in the human eye (1). These different photoreceptors differ in many respects: their wavelength tuning (spectral sensitivity), their temporal properties, their operating range and their distribution across the retina. The goal of this primer is to describe the method of silent substitution for examining photoreceptor-specific pupil responses. We start with the fundamentals underlying the method of silent substitution, provide an overview of studies that have used this method, provide a practical guide and *R* code to implement silent substitution and highlight a few challenges to the method of silent substitution.

## Fundamentals

### Overlapping spectral sensitivities of the human photoreceptors

**Figure 1:**
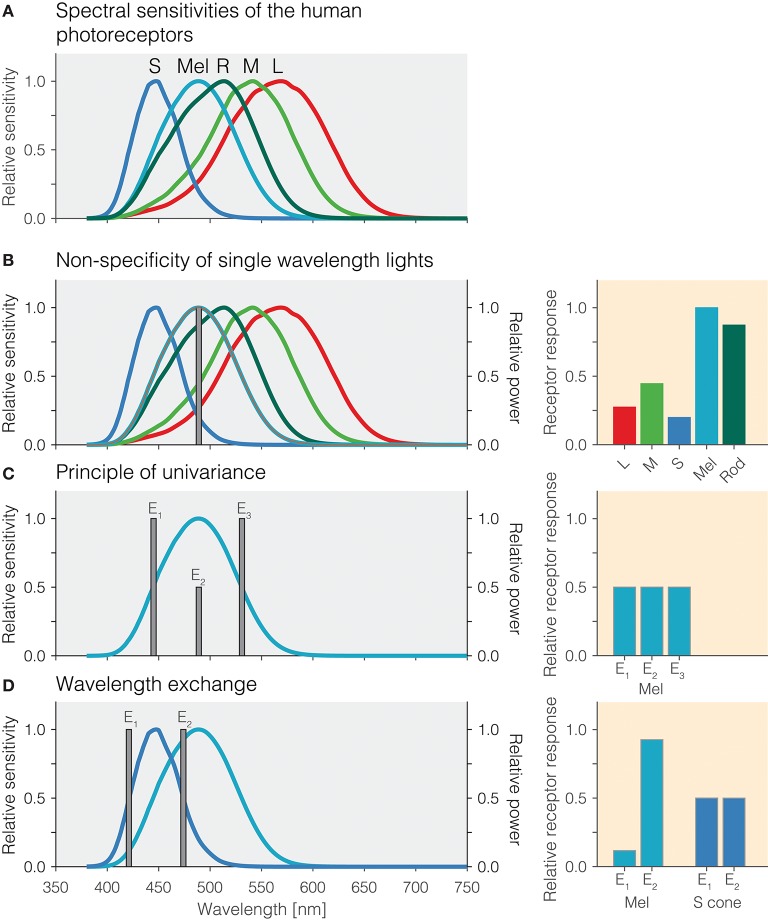
**(A)** Overlapping spectral sensitivities of the human photoreceptors. **(B)** Non-specificity of single-wavelength lights. Right panel: Pattern of photoreceptor responses to the single-wavelength light at 490 nm. **(C)** Principle of univariance. Right panel: Pattern of photoreceptor responses to the single-wavelength lights E_1_, E_2_, and E_3_ designed to elicit the same response in melanopsin. **(D)** Wavelength exchange between two short-wavelength lights E_1_ and E_2_ which stimulate S cones at the same level but yield different photoreceptor responses for melanopsin. Right panel: Pattern of excitations for lights E_1_ and E_2_.

Photoreception in the human retina is based on the signals produced by the three types of cones—the long[L]-wavelength-sensitive cones, the medium[M]-wavelength-sensitive cones, and the short[S]-wavelength-sensitive cones—, the rods, and the intrinsically photosensitive retinal ganglion cells (ipRGCs), which contain the photopigment melanopsin (2–6). ipRGCs receive synaptic input from cones and rods but, in the absence of those inputs, these cells themselves are photosensitive due to the expression of the melanopsin photopigment in the cell membrane. The peak spectral sensitivities (λ_max_) of the human photoreceptors are distinct. The photopigments (cone opsins) in the L, M, and S cones peak around 420, 530, and 558 nm, respectively; rhodopsin, the pigment in rods, has a peak at around 495 nm. Finally, the melanopsin photopigment has a peak spectral sensitivity at around 480 nm. Even though these peaks are spectrally distinct and distant, the spectral sensitivities overlap quite extensively due to the relative broadband tuning of photopigments (Figure 1A). One challenge in targeting the operation of a single class of photoreceptor is that the spectral sensitivities of the photoreceptors *in vivo* does not necessarily correspond to the spectral sensitivity of a pigment. All light that reaches the retina is filtered by the lens and ocular media (7), thereby shifting the effective spectral sensitivity. Typically, this pre-receptoral filtering is accounted for in the spectral sensitivities for cones, rods, and melanopsin-containing ipRGCs.

### Non-specificity of single-wavelength lights

An important desideratum for examining how the different photoreceptors contribute to the human pupillary light response is that stimuli produce responses specific to a given photoreceptor class. One consequence of the extensive spectral overlap of the photoreceptors is that most light sources activate all photoreceptors, and therefore, the responses elicited are largely nonspecific. For example, monochromatic light with a peak spectral output of 490 nm will activate melanopsin maximally relative to the other photoreceptors, but it will also lead to substantial activation of rods and the cones (Figure 1B). The relative amounts by which a monochromatic light of a given wavelength activates all photoreceptors is directly predicted from the relative spectral sensitivity of the photoreceptors at that wavelength. Monochromatic lights have been of great use in determining the spectral sensitivity of the sustained pupil constrictions that match that of melanopsin (8–11). This specific type of measurement is called the “post-illumination pupil response,” abbreviated PIPR, in which the pupil response is typically measured in response to a non-specific short-wavelength light flash and a non-specific long-wavelength light flash against a dim or no background.

### Principle of univariance

One property of photoreceptors is the *principle of univariance* (12), which states that a given photoreceptor has only scalar output, namely its photocurrent: It cannot distinguish between changes in intensity and changes in wavelength. This is shown in Figure 1C using theoretical lights containing power at only a single wavelength (monochromatic light): Lights E_1_, E_2_, and E_3_ all nominally elicit the same photoreceptor excitation. Lights E_1_ and E_3_ have their peak emission at the 50% point of spectral sensitivity on either side of the peak; light E_2_ is scaled to be 50% of the emission of lights E_1_ and E_3_. To the photoreceptor (in this case melanopsin), which weights the input light by its spectral sensitivity, the lights are equally effective. The key insight is that photoreceptors integrate light of different wavelengths, weighting the input spectrum by their spectral sensitivity and summing it up. A consequence of the principle of univariance is that single photoreceptors are color-blind: Whether two lights differ in wavelength or intensity cannot be determined from the photoreceptor output alone.

### Wavelength exchange

Because photoreceptors weight input light by their spectral sensitivity function, in the case of two photoreceptors, it is possible to find two lights and scale them such that the excitation of one of the photoreceptors remains constant in this wavelength exchange, while the other one “sees” a difference. This is shown in Figure 1D: The peak emissions of lights E_1_ and E_2_ have been chosen to match the two 50% points of the S-cone spectral sensitivity, thereby eliciting the same responses. This is called *silencing* the S cones. Because the spectral sensitivity of melanopsin is different from that of the S cones, our two lights E_1_ and E_2_ necessarily produce a different response, and in this case we call melanopsin the *stimulated* photoreceptor. Wavelength exchange for two photopigments is the most simple case of silent substitution. But, with the exception of certain classes of color-blindness such as rod monochromacy, the human retina contains five photoreceptors. Fortunately, the same principle can be extended to more than two photoreceptors.

## The method of silent substitution

In the method of silent substitution, pairs of light are found that have the property that they stimulate the targeted photoreceptor class (or classes) whilst not changing the excitation of the other photoreceptors, the silenced ones. The method has a long history for determining the properties of the mechanisms of human color vision (13, 14).

### Fundamentals

To introduce the method of silent substitution we begin with an example from human color vision. Human color vision is trichromatic under daylight conditions, i.e., when rods do not participate: A color-normal observer can match the color appearance of any light using a combination of three primary lights (15). Under these conditions, it is assumed that only the three classes of cones participate in the color match; it follows that because three photoreceptors participate, three independent primary lights need to be used. It is impossible to match the activation of three photoreceptors in one condition using just two primary lights.

In general, to stimulate one class of photoreceptor classes out of *N*_*R*_ photoreceptor classes while leaving the activation of the other *N*_*R*_−1 unchanged, at least *N*_*R*_ primary lights (*N*_*p*_) are necessary. When *N*_*R*_ = *N*_*P*_ (i. e. there are as many primary lights as photoreceptor classes under consideration), there is only one algebraic solution to match the activation of the *N*_*R*_−1 photoreceptors under one set of settings for the *N*_*P*_ lights to another other setting that will only stimulate the remaining photoreceptor class.

For the case of four photoreceptor classes in the human retina (three classes of cones and melanopsin), four lights are necessary to match the activation of cones and stimulate melanopsin. When including the rods, five lights are necessary to match the activation of cones and rods and stimulate melanopsin.

It is possible to have more primary lights than photoreceptors under consideration, i.e., *N*_*R*_ < *N*_*P*_. This would for example be the case when there were, e.g., eight independent primaries and the retina to be studied was a human one. In that case, there are infinitely many solutions to match the activation of the *N*_*R*_−1 photoreceptors under one set of settings for the *N*_*P*_ lights to any other setting that will only stimulate the remaining photoreceptor class. In practice, this is typically solved by implementing a numerical optimization which maximizes the contrast seen by the stimulated photoreceptor while setting a constraint to have no contrast on the unstimulated ones, and enforcing additional constraints on the optimisation.

#### Contrast

The term contrast refers to a specific quantity, which is the fractional difference of activation of a photopigment around a background:

$$\begin{array}{l} I=\;\frac{I_{modulation}-I_{background}}{I_{background}} \end{array}$$

Intuitively, when the light-adapted background activates a given photoreceptor by some amount, e.g., 100 (arbitrary units), and the modulation activates it by a higher amount, e.g., 120 (arbitrary units), the contrast in that case would be 0.2 or 20%. Contrast can be specified either as fractions or as percentages.

### An intuitive example

We now describe an example case for the method of silent substitution corresponding to the stimuli used in Spitschan, Jain, Brainard and Aguirre (16). These authors used a calibrated spectrally tuneable light source that modified the output of a broadband Xenon arc lamp using a digital micromirror device (DMD) to produce, effectively, arbitrary spectral power distributions. While this is a special case of light sources, most experimenters have used a set of discrete lights, the intensities of which are controlled to produce silent substitution stimuli. *The goal is to produce two lights with spectral power distributions that do not differ in the amount they activate the cones, and only yield a change in the amount they activate melanopsin*. Such pairs of stimuli are also called metamers–they are indistinguishable to the cones, despite having different spectral power distributions. In this example, we ignore the rods.

**Figure 2:**
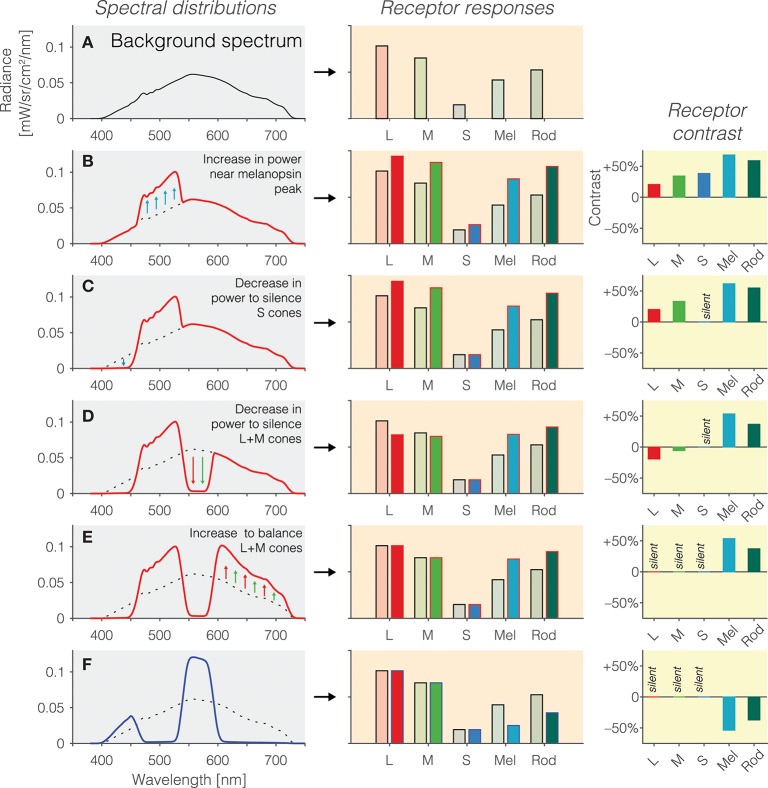
**(A)** Background spectrum (left panel) to which the observer is light-adapted, eliciting a pattern of responses in the photoreceptors (right panel). **(B)** Increase in emitted light near the melanopsin peak relative to the background spectrum (left panel; dashed line = background spectrum, red line = modulation spectrum) leads to an increase in the excitation of all photoreceptors (middle panel), or equivalently, positive contrast on the photoreceptors (right panel). **(C)** To balance the excitation of the S cones, a decrease in emitted short-wavelength light (left panel) leads to silencing of the S cones (middle panel), or equivalently, zero contrast on the S cones (right panel). **(D)** To balance the excitation of the L and M cones, a decrease in emitted medium-wavelength light (left panel) leads to a reduction in L and M cone activity (middle panel) but not yet zero contrast on the L and M cones (right panel); indeed, the contrast seen by the L and M cones is now negative. **(E)** To silence the excitation of the L and M cones, a decrease in emitted long-wavelength light (left panel) leads to balancing of the L and M cones (middle panel), or equivalently, zero contrast on the L and M cones (right panel). The contrast seen by melanopsin is 50%. **(F)** The modulation spectrum shown in **(E)** yields positive contrast relative to the background spectrum but the spectrum can also be “mirrored” around the background spectrum, thereby leading to a negative modulation of melanopsin (and rods).

1. Background spectrum: In the first instance, we begin with a background spectrum of known spectral power distribution (Figure 2A). We call this the background spectrum because the observer is typically light-adapted to this spectrum, and the silent-substitution stimuli are shown to the observer “around” this background in the form of pulses or temporal modulations. This background spectrum elicits a pattern of photoreceptors responses (Figure 2B, right panel). The activation of photoreceptors is calculated by weighting the spectrum by the spectral sensitivities and summing it up for each photoreceptor class.
2. Increasing melanopsin activation: Pragmatically, we can increase the amount of light seen by melanopsin by simply increasing the amount of light emitted near the melanopsin peak. This is shown in Figure 2B. However, this is only partly successful: Because of the overlapping peak spectral sensitivities of the human photoreceptors, such an increase in emitted light leads to an increase in activation of all photoreceptors (Figure 2B, middle panel). Rather than considering the absolute amount of activation of the photoreceptors (which is also dependent on the exact light level), it is customary to speak of contrast (Figure 2B, right panel). Contrast here refers to the percentage difference in activation of photoreceptors between the modulation spectrum (red line in Figure 2B) and the background spectrum (Figure 2A and dashed line in Figures 2B–F). As can be seen in the right panel in Figure 2B, the increase in light near the melanopsin peak leads to an increase in contrast to all photoreceptors. To reiterate, the desideratum here is to have no contrast seen by L, M and S cones, and positive contrast seen by melanopsin.
3. Silencing S cones: To zero, or silence, the S cones, we decrease the amount of short-wavelength light, to which the S cones are most sensitive (Figure 2C). This indeed leads to a silencing of S cones (Figure 2C, middle panel). There is no difference in the absolute activation of S cones, and consequently, the S cone contrast is zero–they are silent.
4. Silencing L and M cones: To silence the L and M cones, a similar trick is applied: Light near the peak spectral sensitivity of L and M cones is decreased to reduce the overall absolute activation of L and M cones (Figure 2D). However, we note that there has been an “overshoot” in the decrease in L and M cones activation (Figure 2D, middle panel): The modulation spectrum is now producing *less* activation in the L and M cones than the background spectrum. This translates into a small amount of negative contrast seen by the L and M cones (Figure 2D, right panel). This can be overcome by again increasing the amount of long-wavelength light in the modulation spectrum (Figure 2E), thereby equalizing the activation of L and M cones relative to the background spectrum (Figure 2D, middle panel). The L, M and S cones are now silent (Figure 2E, right panel), and melanopsin is stimulated at 50%. Because no attempt was taken to silence the rods, they are also stimulated by this spectral exchange.
5. Inverting the melanopsin activation: The modulation spectrum shown in Figure 2E (red line) produces a significant increase in melanopsin excitation. By “mirroring” the modulation spectrum around the background spectrum (i.e., an increase in emitted light in the positive modulation spectrum becomes a decrease by the same amount in emitted light in the negative modulation spectrum), we can also generate a negative (rather than a positive) melanopsin stimulus (Figure 2F, blue line), thereby producing negative, or decremental, contrast on melanopsin (Figure 2F, right panel). In practice, negative and positive modulation spectra are alternated to yield the highest differential activation possible.

### A quantitative example

We provide a quantitative example along with code in Appendices A1, A2, respectively. We use the stimuli from Woelders et al. (17) for this example.

### History of silent substitution

The method of silent substitution has enjoyed use in empirical work well before the discovery of melanopsin and the ipRGCs. We point the reader to Estévez and Spekreijse (13) for an exposé of the early history of the method, which indeed dates back to experiments involving wavelength exchanges performed in 1906 (18) (see above section “Wavelength exchange”). From the insight that metameric lights such as those obtained in color matching experiments are silent substitution stimuli (i.e., matching the activation of the three cone types), the extension to experimentally control more photoreceptors is conceptually straightforward. In the 1990s, methods to manipulate four photoreceptors independently (three cone classes and rods) using mixtures of four primary lights were developed (18, 19). These methods were then expanded to examining melanopsin function either by assuming rod saturation at high light levels (20), or using five primaries (21).

## Overview of silent substitution studies concerning the pupil

We provide an overview of extant studies examining specifically melanopsin photoreception using the method of silent substitution in Table 1 and hope that it serves to the reader as an orientation to the literature. This overview includes literature available in early September 2018. We note that both authors of this article have published papers using the method of silent substitution which are included in the table [M.S.: (16, 28), T.W.: (17)]. The table shows that there is a set of experimental parameters that are subject to the experimenters' discretion. We summarise these here.

**Table 1:** Studies examining human pupil responses with silent substitution.

**References**	**Number of primaries**	**Primary wavelengths [nm]**	**Viewing geometry**	**Field size**	**Modulations [max. contrast]**	**Background light level**	**N observers**	**Temporal properties**	**Spectral sensitivities assumed**
Tsujimura et al. (20)	4	470, 500, 525, 615 nm ± 20–36 nm	Diffusing screen in front of integrating sphere	20° field size	Melanopsin [−53%] Luminance [−53%] Isochromatic [−53%]	301 cd/m^2^ to 642 cd/m^2^ to 982 cd/m^2^	6	10 min stimuli 5 min background	Cones: CIEPO2006 Melanopsin: Dartnall nomogram at 482 nm Assumes macular and lens filtering from CIEPO2006 Optical density 0.5
Viénot et al. (21)	5	473 ± 25 nm 511 ± 33 nm 530 ± 36 nm 595 ± 15 nm 627 ± 20 nm	Light booth with white paint	Ganzfeld	Melanopsin-only (cone and rod silent) [3.4%] Multiple mixed modulations	35 cd/m^2^	10	Measurement after 1 min of continuous exposure	Cones: CIEPO2006 Rods: V'(λ) Melanopsin: Stockman-Sharpe shifted to peak at 482 nm; optical density 0.1; lens from Stockman & Sharpe
Tsijumura and Tokuda, (22)	4	468, 524, 599, and 633 nm (test) 466 nm, 500 nm, 517 nm, 596 nm (background) ±15–38 nm	Diffusing screen in front of integrating sphere	Annulus id 5 od 18° Total field 23°	8%	612 cd/m^2^ background1,109 cd/m^2^ test field	6	Sinusoidal & square wave stimuli	Cones: CIEPO2006, 10° Melanopsin: Dartnall nomogram at 480 nm Assumes macular and lens filtering from CIEPO2006 Optical density 0.4
Spitschan et al. (16)	128	n/a	Viewing of surface through lens	27.5° circular, central 5° blocked	S, (L+M), melanopsin, (L+M+melanopsin) [50%]	382–1,033 cd/m^2^	16	Sinusoidal, 0.01 – 2 Hz	10° Stockman–Sharpe/CIE cone fundamentals, melanopsin estimated by shifting Stockman-Sharpe nomogram to λ_max_ = 480 nm, corrected for prereceptoral filtering (same as cones, optical density 0.3).
Barrionuevo et al. (23)	4	442, 516, 594, and 634 nm (one set) 466, 514, 590, and 634 nm (second set)	Ganzfeld	54° field	Mixed joint modulations, no melanopsin-isolaing modulation	0.002–100 cd/m^2^	3 (authors)	Sinusoidal, 0.5–8 Hz	Smith–Pokorny cone fundamentals Enezi et al. melanopsin function
Cao et al. (25)	5	456, 488, 540, 592, 633	Maxwellian view	30° circular, central 10.5° blocked	Experiment 1: S, M, L, Rod, Melanopsin [16%] Experiment 2: CSF	Experiment 1: 200 Photopic Td Experiment 2: 2,000 Photopic Td	3	Sinusoidal, 1 Hz	Smith–Pokorny cone fundamentals applied for the CIE 1964 10° Standard Observer CIE 1951 scotopic luminosity function Enezi et al. melanopsin function
Barrionuevo and Cao, (24)	5	456, 488, 540, 592, 633	Maxwellian view	30° circular, central 10.5° blocked	Experiment 1: S, M, L, Rods, Melanopsin, (L+M+S) [17%], red-green [4% M,−4% L] Experiment 2: LMS [17% each], L+M [17% each], LMS + melanopsin [16% each], Rods + melanopsin [9% each], S + melanopsin [16% each], red-green + melanopsin [2% M,−2% L, 8% melanopsin]	2–20,000 Photopic Td	3 (2 authors)	Sinusoidal, 1 Hz	Smith–Pokorny cone fundamentals applied for the CIE 1964 10° Standard Observer CIE 1951 scotopic luminosity function Enezi et al. melanopsin function
Spitschan et al. (28)	56	n/a	Viewing of surface through lens	64° circular, central 5° blocked	25–400%	100–200 cd/m^2^	4	Tapered pulses (3 s, 14–16 s ISI)	CIE 2006 parametric model
Woelders et al. (17)	5	465, 500, 515, 595	Diffusing screen in front of LEDs	24.68° horizontal, 12.13° vertical	S, M, L, Melanopsin [23%]	Background or average of 8.5 melanopic lux	16	Square-wave (0.25–4 Hz)	α-opic lux (Lucas et al.): Govardovski nomograms, λ_max_ from Dartnall, optical densities 0.3, 0.38, 0.38 (S, M, L)
Murray et al. (27)	4	460, 524, 590, 635	Ganzfeld	Central 7° of surface covered with disk of no reflective black material.	L, M, (L+M+S) [11% Weber]	17 cd/m^2^	5	Square-wave (1 s increment/ decrement followed by ISI of 2 s)	Stockman Sharpe cone fundamentals
Zele et al. (26)	5	456, 488, 540, 592, 633	Maxwellian view	30° circular, central 10.5° blocked	Color:[7%, 22% or 24% Weber] CFF and pupil: (L+M), S, melanopsin [17% Michelson]	2,000 photopic Td (detection thresholds and pupil) 200–5,000 Td (CFF)	4 (2 authors)	Pupil: 1 Hz sinusoidal	Smith–Pokorny cone fundamentals applied for the CIE 1964 108 Standard Observer CIE 1951 scotopic luminosity function Enezi et al. melanopsin function

### Number of primaries

As described above (*The method of silent substitution–Fundamentals***)**, when stimulating melanopsin, at least four (for matching the cones) or five (for matching both cones and rods) independent primary lights are necessary. Most silent substitution studies that have examined pupil responses to photoreceptor-specific modulations have employed a finite set of LEDs (four or five), though using spectrally tuneable light sources, more effective primaries are possible.

### Peak wavelength and width of the primary lights

In the case where the primary lights are discrete (such as LEDs), the peak emission wavelengths are subject to design considerations when building the apparatus. Both the choice of peak wavelengths and primary widths affects the contrast available for the silent substitution modulations. The contrast available is also called the *gamut*. In principle, choosing broader primaries will reduce also the amount of susceptibility to individual differences in the cone spectral sensitivities (29). In practice, unless a spectrally tuneable light source is used allowing to create arbitrary spectral power distributions, the choices of primary wavelengths and widths is limited by what is commercially available. When building a system, we recommend first estimating the gamut for a given configuration of peak wavelengths and widths.

### Viewing geometry

Typical viewing geometries include Ganzfeld viewing conditions (in which the stimulus is a homogenous field in an integrating sphere) or Maxwellian view (in which an image is focused on the entrance pupil of the observer). These again depend on the type of design used when building the stimulation system.

### Field size

As can be seen in the table, the field sizes used in the field vary somewhat, and will again depend on constraints set by the optical apparatus used to deliver the stimuli, as well as theoretical considerations such as the distribution of the photoreceptor types across the retina.

### Modulations and contrast

Depending on the spectra of the primary lights, different amounts of contrast are available to stimulate melanopsin. Typically, the highest contrast can be achieved when LEDs are chosen of which the distribution of peak wavelengths is as broad as possible.

### Background light

The choice of background light level is again somewhat arbitrary in many situations, though experimenters typically strive to be well in photopic conditions, where rods are assumed to be saturated, and can therefore be ignored (but see *Rod intrusion* below).

### Spectral sensitivities assumed

The extent to which a given melanopsin-stimulating modulation silences the cones depends on the spectral sensitivities assumed. Various spectral sensitivities are available (30). Choosing the wrong spectral sensitivities can lead to artefactual results, unless care is taken to correct the modulations. We recommend the use of the CIE 2006 “physiologically relevant” cone fundamentals (31) as it allows for flexible extensions to simulate individual differences parametrically (32). For melanopsin, there is currently no standard(ised) spectral sensitivity, though by using a template (also called nomogram) centered at 480 nm and assuming a low peak optical density, such a spectral sensitivity can easily be derived (33, 34).

## Challenges to silent substitution

There are various sources of uncertainty when using silent substitution stimuli. We highlight a few of these here.

### Retinal inhomogenities

The human retina is inhomogeneous. One obvious feature of the retina making it inhomogeneous is the spatial location of the macular pigment around the fovea, with a drop-off toward the periphery. A consequence of macular pigment is that all light seen by the fovea is filtered through the pigment, thereby shifting the effective peak spectral sensitivity of the foveal cones vs. the peripheral cones. In addition, there are also differences in how much photopigment is expressed in foveal vs. peripheral cones—the optical densities are different. Another source of retinal inhomogeneity is that cones that are in the partial shadow of retinal blood vessels—penumbral cones—have a different spectral sensitivity than the open-field cones (35). Effectively, for the method of silent substitution, this means that that there are three additional photoreceptor classes that need to be silenced, and therefore, more primaries are necessary. Practically, penumbral cones can be desensitized using a white-noise stimulus (26), or silenced, though with a significant drop in contrast (36).

### Individual differences in the cone spectral sensitivities

There are individual differences in the spectral sensitivities of the cones and this biological variability will affect the degree to which the cones are truly silenced in a melanopsin-directed modulation. Inter-observer differences have been a concern in the accurate specification of cone signals well before the discovery of melanopsin (37–39). Biological variability arises from inter-observer variability in lens density, macular pigment density, taxial density of the pigment (32, 37–39); and the peak spectral sensitivity due to polymorphisms in the opsin genes (40–43). A given set of cone fundamentals only describes the average spectral sensitivities within a population and ignoring biological variability will introduce error. In the field of melanopsin-mediated pupillometry, some experimenters correct the stimuli by having the observers perform a color matching procedure (25, 26), while others (16, 17, 28) simulate the variability of the stimuli using simulations based on estimates of the biological variability of parameters of the cones (32).

### Melanopsin bistability and tristability

The method of silent substitution assumes that melanopsin the spectral sensitivity of melanopsin can be described by a single function. There is ample evidence that melanopsin is a bistable (44–49) or tristable photopigment (50). While the cone and rod photopigment is regenerated in the retinal pigment epithelium (RPE), melanopsin, being expressed in ipRGCs in the inner retina, and therefore removed from the RPE is thought to rely on a different mechanism for pigment regeneration. A bistable (or tristable) photopigment relies on light itself to regenerate the pigment, and that this regeneration process again is wavelength-dependent and therefore has a separate spectral sensitivity. It is controversial whether the multistable photochemistry of melanopsin has physiological consequences (51–53). Under conditions of adaptating to a constant background light as employed in silent substitution, melanopsin will be in photoequlibrium, i.e., the different states of the pigment will exist in fixed (though possibly unknown) proportions.

### Rod intrusion

Under daylight conditions, rods are typically thought to be saturated (54, 55), though the range of light levels in which both rods and cones are known to be active is substantial (56, 57). Using a five-primary stimulator [e.g., (25, 26)], it is possible to generate melanopsin-directed stimuli which not only silence the cones but also silence rods. Typically, when rods are silenced, the contrast available to melanopsin is typically only around 1/3 relative to a stimulus in which rods are ignored, though this will depend on the choice of background.

### Scatter

The human eye is an imperfect optical system. In cases where the stimulus is a spatially extended light source and there is light outside the primary stimulation area (both centrally, if the macular region is blocked, and in the far periphery), there will be undesired stimulation of potentially unadapted photoreceptors (such as the rods). This can be addressed by adding a light outside the primary stimulation area that light-adapts the photoreceptors outside of the primary stimulation area.

### Device uncertainty

The light source used may not be stable over time and change spectral output between operations, or throughout the sessions. These drifts in device output need to be either calibrated, or at least characterized.

## Exploiting properties other than spectral sensitivity

We have noted in the introduction that the photoreceptors contributing to pupillary control differ not only in their spectral sensitivity (as is exploited in the method of silent substitution) but also in their temporal properties, their operating range and their distribution across the retina. These properties might also be exploited to selectively stimulate melanopsin. For example, the retinal location corresponding to the blind spot does not contain rods and cones, but light might stimulate melanopsin in the axons of ipRGCs. Delivering a stimulus only in the blind spot would therefore ensure that only melanopsin would be activated (58–60), but there could be scatter on rod and cone photoreceptors near the blind spot, and accidental displacement of a small circumscribed stimulus field would need to be controlled for. In the temporal domain, melanopsin photoreception is much slower than cone- and rod-mediated photoreception, and thus, the temporal properties of a stimulus can be optimized to bias the measured response toward melanopsin-mediated properties, e.g., the steady-state pupil size under continuous light (61).

## Conclusion

The method of silent substitution is a powerful technique to stimulate a specific photoreceptor class or specific photoreceptor classes in the living human retina while leaving other classes un-stimulated. The method has been used successfully to examine the photoreceptor contributions to the human pupillary light responses. The method is not failsafe as several factors need to be considered (retinal inhomogeneities, individual differences, rod intrusion, scatter, and device uncertainty), but these can be addressed experimentally or in simulation. We hope that the method of silent substitution will gain traction to tease apart the contributions of different photoreceptors to human vision and to elucidate their role in the non-invasive assessment of the human visual system using pupillometry.

## Author contributions

All authors listed have made a substantial, direct and intellectual contribution to the work, and approved it for publication.

### Conflict of interest statement

The authors declare that the research was conducted in the absence of any commercial or financial relationships that could be construed as a potential conflict of interest.
