# Supplementary material for: The Method of Silent Substitution for Examining Melanopsin Contributions to Pupil Control
Source: Front Neurol. 2018 Nov 27;9:941. doi: 10.3389/fneur.2018.00941 (PMC6277556; doi:10.3389/fneur.2018.00941)
Supplement: Supplementary file 1 [file Data_Sheet_1.pdf]

# Appendix A1: Tutorial

This tutorial aims to provide a guide in designing pairs of silent substitution spectra, in this case melanopsin-isolating stimuli. The tutorial is supported by Appendix A2, which contains annotated *R*-code that can be used to perform all calculations discussed in this tutorial. For a MATLAB-based solution, please consult the Silent Substitution Toolbox at https://github.com/spitschan/SilentSubstitutionToolbox.

**Source**

The stimuli described here are those used in Woelders T, Leenheers T, Gordijn MCM, Hut RA, Beersma DGM, Wams EJ. Melanopsin- and L-cone-induced pupil constriction is inhibited by S- and M-cones in humans. *Proc Natl Acad Sci USA*. (2018) 115:792–7. doi: 10.1073/pnas.1716281115.

For more information about the paradigm, please consult the paper. Note that this tutorial does not provide a normative solution; all the caveats listed in the main manuscript apply.

**Objective**

We have access to a five-primary light source with peak wavelengths of 465 (“blue”), 500 (“cyan”), 515 (“green”), 595 (“yellow”), and 635 nm (“red”) where the intensities of the individual LEDs can be controlled at 8-bit resolution (0-255 intensity range). Our goal is to find the LED settings (intensities for blue, cyan, green, yellow and red LEDs) required to produce two spectra that differ in melanopic illuminance, but are equal in terms of S-, M- and L-cone-specific illuminances. Furthermore, we want to explore the range of melanopic illuminance Michelson contrasts achievable with our device at a background of 1 melanopic lux. Finally, we will design a pair of silent substitution spectra that modulates melanopic illuminance at a pre-defined Michelson contrast that is within this range.

**Spectral characteristics of the light source**

We have spectral measurements of each LED in our light source. From the spectral measurements, we have obtained the photoreceptor-specific illuminance values for each LED set to maximum intensity (Table A1).

| | **Blue** | **Cyan** | **Green** | **Yellow** | **Red** |
|---|---|---|---|---|---|
| **S** | 58.54 | 1.81 | 0.85 | 0.05 | 0.07 |
| **M** | 16.51 | 7.96 | 12.67 | 10.1 | 5.61 |
| **L** | 8.65 | 5.14 | 9.13 | 16.91 | 19.99 |
| **Mel** | 47.5 | 12.18 | 13.08 | 0.33 | 0.09 |

**Table A1. Photoreceptor-specific illuminance values (lux) for each LED at maximum intensity.**

There exists a linear relationship between the intensity of each LED and its photoreceptor-specific illuminance values. Therefore, dividing all values in Table A1 by 255 (maximal intensity setting) provides the slope coefficients for the relationship between intensity and photoreceptor-specific illuminance values for each LED separately (Table A2). For example, a 1-step increment in intensity for the blue LED is associated with an increase of 0.23, 0.06, 0.03 and 0.19 S-cone, M-cone, L-cone and melanopic lux respectively.

| | Blue | Cyan | Green | Yellow | Red |
|---|---|---|---|---|---|
| **S** | 0.229568627 | 0.007098039 | 0.003333333 | 0.000196078 | 0.00027451 |
| **M** | 0.064745098 | 0.031215686 | 0.049686275 | 0.039607843 | 0.022 |
| **L** | 0.033921569 | 0.020156863 | 0.035803922 | 0.066313725 | 0.078392157 |
| **Mel** | 0.18627451 | 0.047764706 | 0.051294118 | 0.001294118 | 0.000352941 |

**Table A2. Slope-coefficients for photoreceptor-specific illuminance values (lux) for each LED.**

**Mathematical denotations**

For simplicity, we will denote the slope values as '$LED_r$' where 'LED' indicates the colour of the LED (B, C, G, Y or R) and the subscript indicates the relevant receptor (Table A3).

| | Blue | Cyan | Green | Yellow | Red |
|---|---|---|---|---|---|
| **S** | $B_S$ | $C_S$ | $G_S$ | $Y_S$ | $R_S$ |
| **M** | $B_M$ | $C_M$ | $G_M$ | $Y_M$ | $R_M$ |
| **L** | $B_L$ | $C_L$ | $G_L$ | $Y_L$ | $R_L$ |
| **Mel** | $B_{Mel}$ | $C_{Mel}$ | $G_{Mel}$ | $Y_{Mel}$ | $R_{Mel}$ |

**Table A3. Mathematical denotation of slope coefficients.**

Finally, we will denote the intensity settings of the LEDs as '$I_{LED}$' where 'LED' indicates the colour. $I_B$, $I_C$, $I_G$, $I_Y$ and $I_R$ can take any value in the 0-255 range. When multiplying a 1 x 5 LED intensity matrix [$I_B$, $I_C$, $I_G$, $I_Y$ $I_R$] by the transpose of the 4 x 5 slope coefficient matrix (Table A2), the result is a 1 x 4 matrix containing the photoreceptor-specific illuminance values. Table A4 illustrates how this matrix multiplication yields the four photoreceptor-specific illuminance values.

| | Blue | Cyan | Green | Yellow | Red | Σ |
|---|---|---|---|---|---|---|
| **S** | $I_B$ x $B_S$ | $I_C$ x $C_S$ | $I_G$ x $G_S$ | $I_Y$ x $Y_S$ | $I_R$ x $R_S$ | S-cone lux |
| **M** | $I_B$ x $B_M$ | $I_C$ x $C_M$ | $I_G$ x $G_M$ | $I_Y$ x $Y_M$ | $I_R$ x $R_M$ | M-cone lux |
| **L** | $I_B$ x $B_L$ | $I_C$ x $C_L$ | $I_G$ x $G_L$ | $I_Y$ x $Y_L$ | $I_R$ x $R_L$ | L-cone lux |
| **Mel** | $I_B$ x $B_{Mel}$ | $I_C$ x $C_{Mel}$ | $I_G$ x $G_{Mel}$ | $I_Y$ x $Y_{Mel}$ | $I_R$ x $R_{Mel}$ | Melanopic lux |

**Table A4. Calculation of photoreceptor-specific illuminance values.**

**Producing $Spec_{min}$ and $Spec_{max}$ with the highest melanopic illuminance contrast**

We denote the spectrum with the lowest and highest melanopic illuminance as $Spec_{min}$ and $Spec_{max}$ respectively, and the associated melanopic illuminance values as $Mel_{min}$ and $Mel_{max}$. As per our objective, we first want to maximize $Mel_{max}$ in $Spec_{max}$ at a background $Mel_{min}$ of 1 melanopic lux in $Spec_{min}$, while maintaining identical photoreceptor-specific illuminance values for the cones between $Spec_{min}$ and $Spec_{max}$. This involves a constrained optimization problem in a system of linear equations, as illustrated in Table A5. Note that for this procedure, we extended the intensity denotations so that we have $I_{B0}$, $I_{C0}$, $I_{G0}$, $I_{Y0}$ and $I_{R0}$ producing the $Spec_{min}$ spectrum and $I_{B1}$, $I_{C1}$, $I_{G1}$, $I_{Y1}$ and $I_{R1}$ producing the $Spec_{max}$ spectrum.

| Spec$_{min}$ | | | | | Spec$_{max}$ | | | | | Σ |
|---|---|---|---|---|---|---|---|---|---|---|
| $I_{B0}B_S$ | $I_{C0}C_S$ | $I_{G0}G_S$ | $I_{Y0}Y_S$ | $I_{R0}R_S$ | $-I_{B1}B_S$ | $-I_{C1}C_S$ | $-I_{G1}G_S$ | $-I_{Y1}Y_S$ | $-I_{R1}R_S$ | = 0 |
| $I_{B0}B_M$ | $I_{C0}C_M$ | $I_{G0}G_M$ | $I_{Y0}Y_M$ | $I_{R0}R_M$ | $-I_{B1}B_M$ | $-I_{C1}C_M$ | $-I_{G1}G_M$ | $-I_{Y1}Y_M$ | $-I_{R1}R_M$ | = 0 |
| $I_{B0}B_L$ | $I_{C0}C_L$ | $I_{G0}G_L$ | $I_{Y0}Y_L$ | $I_{R0}R_L$ | $-I_{B1}B_L$ | $-I_{C1}C_L$ | $-I_{G1}G_L$ | $-I_{Y1}Y_L$ | $-I_{R1}R_L$ | = 0 |
| $I_{B0}B_{Mel}$ | $I_{C0}C_{Mel}$ | $I_{G0}G_{Mel}$ | $I_{Y0}Y_{Mel}$ | $I_{R0}R_{Mel}$ | | | | | | = 1 |
| $I_{B0}B_{Mel}$ | | | | | $I_{B1}B_{Mel}$ | $I_{C1}C_{Mel}$ | $I_{G1}G_{Mel}$ | $I_{Y1}Y_{Mel}$ | $I_{R1}R_{Mel}$ | max |

**Table A5. Optimization problem for producing maximal melanopic illuminance Michelson contrast at a background of 1 melanopic lux.**

Note that a linear summation of the 10 first elements in the top row only meets the required constraint of '=0' when the S-cone illuminance produced by $I_{B0}$, $I_{C0}$, $I_{G0}$, $I_{Y0}$ and $I_{R0}$ is identical to the S-cone illuminance produced by $I_{B1}$, $I_{C1}$, $I_{G1}$, $I_{Y1}$ and $I_{R1}$. The same is true for the second and third rows, which are associated with M-cone and L-cone illuminances respectively. A background $Mel_{min}$ of 1 melanopic lux is produced when the linear summation of the five elements in the fourth row equals 1. Finally, the maximal $Mel_{max}$ that is possible at a background of 1 melanopic lux can be found by maximizing the linear summation of the 5 elements in the fifth row. To summarize, solving this problem results in a set of five LED intensity values that produces a $Spec_{min}$ with a $Mel_{min}$ of 1 melanopic lux and a different set of five LED intensity values that produces the highest possible $Mel_{max}$ in $Spec_{max}$ with the constraint that the photoreceptor-specific illuminance values for the cones do not differ between $Spec_{min}$ and $Spec_{max}$. We solve this linear optimization problem using the '*lpSolveAPI*' package in *R*, to obtain the $I_{B0}$, $I_{C0}$, $I_{G0}$, $I_{Y0}$ and $I_{R0}$ necessary to produce the $Spec_{min}$ spectrum and the $I_{B1}$, $I_{C1}$, $I_{G1}$, $I_{Y1}$ and $I_{R1}$ necessary to produce the $Spec_{max}$ spectrum. The resulting intensity settings are provided in Table A6.

| | $I_B$ | $I_C$ | $I_G$ | $I_Y$ | $I_R$ |
|---|---|---|---|---|---|
| $Spec_{min}$ | 3.596842 | 0 | 0 | 255 | 0 |
| $Spec_{max}$ | 0 | 73.941053 | 92.414778 | 0 | 156.045958 |

**Table A6. Intensity settings producing $Spec_{min}$ and $Spec_{max}$ with maximal melanopic illuminance Michelson contrast.**

The photoreceptor-specific illuminance values can now be calculated separately for $Spec_{min}$ and $Spec_{max}$ from the obtained intensity settings, according to the matrix multiplication procedure described in Table A4. The resulting photoreceptor-specific illuminance values for the two spectra are presented in Table A7.

| | S | M | L | Mel |
|---|---|---|---|---|
| $Spec_{min}$ | 0.8757219 | 10.33288 | 17.03201 | 1 |
| $Spec_{max}$ | 0.8757219 | 10.33288 | 17.03201 | 8.327182 |

**Table A7. Photoreceptor-specific illuminance values for $Spec_{min}$ and $Spec_{max}$ with maximal melanopic illuminance Michelson contrast.**

From these calculations, it follows that the maximal Michelson contrast that can be achieved when assuming a background $Mel_{min}$ of 1 melanopic lux is (8.327182 - 1) / (8.327182 + 1) x 100 = 78.5%, by using the intensity settings provided in Table A7. The procedure can be repeated for different values of $Mel_{min}$ to explore the maximal possible contrast under different background conditions for our specific light source.

**Producing $Spec_{min}$ and $Spec_{max}$ with a pre-defined melanopic illuminance contrast**

In the previous example, we found the maximal Michelson contrast possible when a background of 1 melanopic lux is assumed (78.5%). It is also possible and often necessary to solve the problem for a pre-determined contrast of, for example, 50% Michelson contrast. To this end, we add an additional constraint on $Spec_{max}$ so that its melanopic illuminance is 3 melanopic lux ([3 – 1] / [3 + 1] x 100 = 50%). The system of linear equations to be solved in this case can be found in Table A8. The resulting intensity settings are provided in Table A9 and the photoreceptor-specific illuminance values can be found in Table A10.

| **$Spec_{min}$** | | | | | **$Spec_{max}$** | | | | | **Σ** |
|---|---|---|---|---|---|---|---|---|---|---|
| $I_{B0}B_S$ | $I_{C0}C_S$ | $I_{G0}G_S$ | $I_{Y0}Y_S$ | $I_{R0}R_S$ | $-I_{B1}B_S$ | $-I_{C1}C_S$ | $-I_{G1}G_S$ | $-I_{Y1}Y_S$ | $-I_{R1}R_S$ | = 0 |
| $I_{B0}B_M$ | $I_{C0}C_M$ | $I_{G0}G_M$ | $I_{Y0}Y_M$ | $I_{R0}R_M$ | $-I_{B1}B_M$ | $-I_{C1}C_M$ | $-I_{G1}G_M$ | $-I_{Y1}Y_M$ | $-I_{R1}R_M$ | = 0 |
| $I_{B0}B_L$ | $I_{C0}C_L$ | $I_{G0}G_L$ | $I_{Y0}Y_L$ | $I_{R0}R_L$ | $-I_{B1}B_L$ | $-I_{C1}C_L$ | $-I_{G1}G_L$ | $-I_{Y1}Y_L$ | $-I_{R1}R_L$ | = 0 |
| $I_{B0}B_{Mel}$ | $I_{C0}C_{Mel}$ | $I_{G0}G_{Mel}$ | $I_{Y0}Y_{Mel}$ | $I_{R0}R_{Mel}$ | | | | | | = 1 |
| $I_{B0}B_{Mel}$ | | | | | $I_{B1}B_{Mel}$ | $I_{C1}C_{Mel}$ | $I_{G1}G_{Mel}$ | $I_{Y1}Y_{Mel}$ | $I_{R1}R_{Mel}$ | = 3 |

**Table A8. Calculation of photoreceptor-specific illuminance values for a pre-defined melanopic illuminance Michelson contrast of 50%.**

| | **$I_B$** | **$I_C$** | **$I_G$** | **$I_Y$** | **$I_R$** |
|---|---|---|---|---|---|
| $Spec_{min}$ | 0.6120993 | 0 | 9.4134075 | 255 | 207.1988906 |
| $Spec_{max}$ | 0 | 0 | 52.3001089 | 175.650200 | 255 |

**Table A9. Intensity settings producing $Spec_{min}$ and $Spec_{max}$ with a pre-defined melanopic illuminance Michelson contrast of 50%.**

| | **S** | **M** | **L** | **Mel** |
|---|---|---|---|---|
| $Spec_{min}$ | 0.2787749 | 15.16572 | 33.51057 | 1 |
| $Spec_{max}$ | 0.2787749 | 15.16572 | 33.51057 | 3 |

**Table A10. Photoreceptor-specific illuminance values for $Spec_{min}$ and $Spec_{max}$ with a pre-defined melanopic illuminance contrast of 50%.**

# Appendix A2: *R* code

This file serves as a practical guide to design silent-substitution spectra in *R*. This guide supports the example discussed in Appendix A1 and contains references to a selection of tables displayed in Appendix A1.

## Preparations

First, we create a file called *LED_illuminances.csv* which corresponds to the activation of each photoreceptor in response to the primary lights (corresponds to Table A1). This file needs to be in the working directory.

```
,Blue,Cyan,Green,Yellow,Red
S,58.54,1.81,0.85,0.05,0.07
M,16.51,7.96,12.67,10.10,5.61
L,8.65,5.14,9.13,16.91,19.99
Mel,47.50,12.18,13.08,0.33,0.09
```

Next we load the this file into the *R* workspace:

```
maxIlluminances <- read.csv("LED_illuminances.csv", header=TRUE, stringsAsFac
tors=FALSE)[,2:6]
print(maxIlluminances)

##    Blue  Cyan Green Yellow   Red
## 1 58.54  1.81  0.85   0.05  0.07
## 2 16.51  7.96 12.67  10.10  5.61
## 3  8.65  5.14  9.13  16.91 19.99
## 4 47.50 12.18 13.08   0.33  0.09
```

We divide by 255 to get the slope coefficients (Table A2):

```
coefs <- maxIlluminances/255
print(coefs)

##         Blue        Cyan       Green       Yellow          Red
## 1 0.22956863 0.007098039 0.003333333 0.0001960784 0.0002745098
## 2 0.06474510 0.031215686 0.049686275 0.0396078431 0.0220000000
## 3 0.03392157 0.020156863 0.035803922 0.0663137255 0.0783921569
## 4 0.18627451 0.047764706 0.051294118 0.0012941176 0.0003529412
```

We load the '*lpSolveAPI*' package for solving optimization problems in systems of linear equations:

```
#install.packages(lpSolveAPI) #uncomment to install lpSolveAPI package
library(lpSolveAPI)
```

### *Setup and solve system of linear equations (Table A5) to maximize $Mel_{max}$ at a background of 1 melanopic lux*

We set up the system of linear equations using several *lpSolveAPI* functions. We first recreate the optimization problem illustrated in Table A5:

```
#create a 4-row 10-column system of linear equations to recreate the first fo
ur rows in Table A5
systemLE <- make.lp(4,10)

#add the photoreceptor-specific illuminance coefficients (and their negatives
)
set.row(systemLE, 1, c(coefs[1,], -coefs[1,]), indices=c(1:10))
set.row(systemLE, 2, c(coefs[2,], -coefs[2,]), indices=c(1:10))
set.row(systemLE, 3, c(coefs[3,], -coefs[3,]), indices=c(1:10))
set.row(systemLE, 4, c(coefs[4,]), indices=c(1:5))

#set the right-hand side constraint types and values for the first four rows
set.constr.type(systemLE, c("=","=","=","="))
set.rhs(systemLE, c(0,0,0,1))

#add the fifth row, which contains the photoreceptor-specific illuminance coe
fficients of the linear equation that is maximized
set.objfn(systemLE, c(coefs[4,]), indices=c(6:10))
lp.control(systemLE, sense= "max")

#allow only LED intensity values ranging from 0 to 255
set.bounds(systemLE, lower = rep(0,10))
set.bounds(systemLE, upper = rep(255,10))
```

Now we can solve the problem to find the two sets of five LED intensity values producing $Spec_{min}$ and $Spec_{max}$. The optimizer will find a set of 10 intensity values ($I_{B0}$, $I_{C0}$, $I_{G0}$, $I_{Y0}$, $I_{R0}$, $I_{B1}$, $I_{C1}$, $I_{G1}$, $I_{Y1}$, $I_{R1}$) by which the illuminance coefficients in Table A5 (i.e. the indices in *systemLE*) should be multiplied in order to maximize $Mel_{max}$ while also satisfying the right-hand side summation constraints.

```
#find a solution
solve(systemLE)

#store the solution
LEDIntensities <- get.variables(systemLE)
```

### *Results $Mel_{max}$ maximization (Tables S6 and S7)*

These are the required LED intensity values that will meet our requirements. These 5 LED values ($I_{B0}$, $I_{C0}$, $I_{G0}$, $I_{Y0}$, $I_{R0}$) will produce $Spec_{min}$:

```
LEDIntensities[1:5]
```

```
## [1]   3.596842   0.000000   0.000000 255.000000   0.000000
```

with these S-cone, M-cone, L-cone and melanopic illuminance values:

```
as.numeric(LEDIntensities[1:5] %*% t(coefs)) #matrix-multiplication of LED in
tensities and transpose of illuminance coefficients

## [1]  0.8757221 10.3328779 17.0320105  1.0000000
```

And these 5 LED values ($I_{B1}$, $I_{C1}$, $I_{G1}$, $I_{Y1}$, $I_{R1}$) will produce $Spec_{max}$:

```
LEDIntensities[6:10]

## [1]   0.00000  73.94109  92.41475   0.00000 156.04596
```

with these S-cone, M-cone, L-cone and melanopic illuminance values:

```
as.numeric(LEDIntensities[6:10] %*% t(coefs)) #matrix-multiplication of LED i
ntensities and transpose of illuminance coefficients

## [1]  0.8757221 10.3328779 17.0320105  8.3271828
```

*Setup and solve system of linear equations (Table A8) to find pre-defined melanopic lux contrast of 50% Michelson contrast*

```
#create a 5-row 10-column system of linear equations to recreate the first fi
ve rows in Table A8
systemLE <- make.lp(5,10)

#add the photoreceptor-specific illuminance coefficients (and their negatives
)
set.row(systemLE, 1, c(coefs[1,], -coefs[1,]), indices=c(1:10))
set.row(systemLE, 2, c(coefs[2,], -coefs[2,]), indices=c(1:10))
set.row(systemLE, 3, c(coefs[3,], -coefs[3,]), indices=c(1:10))
set.row(systemLE, 4, c(coefs[4,]), indices=c(1:5))
set.row(systemLE, 5, c(coefs[4,]), indices=c(6:10))

#set the right-hand side values for the first five rows.
set.constr.type(systemLE, c("=","=","=","=","="))
set.rhs(systemLE, c(0,0,0,1,3))

#add a sixth row, which contains the photoreceptor-specific illuminance coeff
icients of the linear equation that is maximized. Note that we have already s
et a constraint on indices 6 to 10 for these coefficients, therefore we alrea
dy know that the outcome of the maximization will be 3 melanopic lux. Because
lpSolveAPI requires an objective function, we add it anyway inspite of its re
dundancy.
set.objfn(systemLE, c(coefs[4,]), indices=c(6:10))
```

```
lp.control(systemLE, sense= "max")

#allow only LED intensity values ranging from 0 to 255
set.bounds(systemLE, lower = rep(0,10))
set.bounds(systemLE, upper = rep(255,10))

#find a solution
solve(systemLE)

#store the solution
LEDIntensities <- get.variables(systemLE)
```

*Results finding pre-defined melanopic lux contrast*

These are the required LED intensity values that will meet our requirements. These 5 LED values ($I_{B0}$, $I_{C0}$, $I_{G0}$, $I_{Y0}$, $I_{R0}$) will produce $Spec_{min}$ :

```
LEDIntensities[1:5]

## [1]   0.6120991   0.0000000   9.4134091 255.0000000 207.1988912
```

with these S-cone, M-cone, L-cone and melanopic illuminance values:

```
as.numeric(LEDIntensities[1:5] %*% t(coefs)) #matrix-multiplication of LED in
tensities and transpose of illuminance coefficients

## [1]  0.2787749 15.1657233 33.5105683  1.0000000
```

And these 5 LED values ($I_{B1}$, $I_{C1}$, $I_{G1}$, $I_{Y1}$, $I_{R1}$) will produce $Spec_{max}$ :

```
LEDIntensities[6:10]

## [1]   0.00000   0.00000  52.30011 175.65020 255.00000
```

with these S-cone, M-cone, L-cone and melanopic illuminance values:

```
as.numeric(LEDIntensities[6:10] %*% t(coefs)) #matrix-multiplication of LED i
ntensities and transpose of illuminance coefficients

## [1]  0.2787749 15.1657233 33.5105683  3.0000000
```
